# Heavy Metals in Harvested Rainwater Used for Domestic Purposes in Rural Areas: Yatta Area, Palestine as a Case Study

**DOI:** 10.3390/ijerph19052683

**Published:** 2022-02-25

**Authors:** Fathi Anabtawi, Nidal Mahmoud, Issam A. Al-Khatib, Yung-Tse Hung

**Affiliations:** 1 Faculty of Graduate Studies, Birzeit University, Birzeit P.O. Box 14, West Bank, Palestine; fathianabtawi@gmail.com; 2 Institute of Environmental and Water Studies, Birzeit University, Birzeit P.O. Box 14, West Bank, Palestine; nmahmoud@birzeit.edu; 3 Department of Civil and Environmental Engineering, Cleveland State University, Cleveland, OH 44115, USA; yungtsehung@gmail.com

**Keywords:** heavy metals, harvested rainwater, health risk, developing countries

## Abstract

Rainwater harvesting is considered one of the most important water resources in the Palestinian countryside. In this research, the study area chosen for the study was Yatta town in Hebron city. 75 water samples were collected from 74 cisterns in a number of neighborhoods in Yatta, and a structured household survey was conducted with the same households where the water samples were collected. Statistical analysis was made using the SPSS software. An analysis for the samples was made using ICP-MS to test the existence of a number of heavy metals, namely Pb, Cr, Mn, Co, Ni, Cu, Zn and Cd. The results were compared with the WHO and Palestinian limits for drinking water quality. Considering the metals Mn, Co, Cu and Cd, neither of the samples exceeded any of the two limits. For the metals, Pb, Cr, and Ni, two samples exceeded both limits. For the metal, Zn, one sample exceeded the WHO limit only. Sources of pollution by heavy metals of the harvested rainwater were identified by means of a questionnaire distributed to the households. The results showed that except for nickel and the water collection surface of the cistern factor, there is no direct relationship between the factors and activities that may contribute to contaminate harvested rainwater with heavy metals and the existence of heavy metals beyond local and international limits. Based on the questionnaire and literature: Possible sources of lead and zinc are the roof, storage tanks, distribution systems and plumbing; possible sources of chromium are road dust, asbestos brakes and anthropogenic activities occurring around the house; possible source of nickel is leaching from metals in contact with harvested rainwater such as pipes and fittings which are used to collect the harvested rainwater. In addition, an assessment of the potential health risks due to contamination of the harvested rainwater by heavy metals was made for all the samples that exceeded either WHO limit or the Palestinian limit or both. The Chronic Daily Intake (CDI) and the Health Risk Index (HRI) were calculated. The assessment was made for both adults and children. The results showed that all the samples are considered safe (HRI < 1), which means that there are no potential health risks for consumers.

## 1. Introduction

Supply of water security that meets the quality parameters specified in applicable standards, is now the basis for the functioning of most societies [1]. Worldwide, one in three people do not have access to safe drinking water, and so billions of people still lack access to clean drinking water [2]. Ensuring access to water and sanitation for all through sustainable management of water resources is the sixth of the 17 goals of the Sustainable Development Goals, developed by the UN General Assembly in 2015 [1]. The climate change impacts on the water resources quantity and quality, besides the increasing frequency of extreme events, e.g., floods and droughts, are becoming an important problem [3]. Water is the most important resource; it is utilized largely in agricultural production and is fundamental to ensuring global food security. The overutilization of water resources creates complex problems, such as waterlogging and salinization, and results in the depletion of groundwater resources [4]. Water is a vital substance in the environment, and its contamination with heavy metals is considered a worldwide environmental problem [5,6,7]. Heavy metals are important pollutants to groundwater, surface water, and harvested rainwater [8,9,10,11]. Common heavy metals that human beings are exposed to include: Aluminum (Al), Cadmium (Cd), Chromium (Cr), Lead (Pb), Mercury (Hg), Copper (Cu), Zinc (Zn), Iron (Fe), Nickel (Ni) and Cobalt (Co).

Among the various pollutants of harvested rainwater, heavy metals are always of big concerns due to their severe toxicities, and so heavy metals are regulated in drinking water by the United States Environmental Protection Agency (U.S. EPA, USA) [12]. When heavy metals enter into the human body, they could easily bind to vital cellular components and accumulate in organisms, resulting in a series of diseases and disorders, e.g., cancers, osteomalacia, kidney malfunction, etc. [13], depending on the type and amount of the metal involved [14,15]. Their toxicity is made by forming complexes with proteins where they contain carboxylic acid (−COOH), amine (−NH_2_) and thiol (−SH) groups.

Worldwide, cancer is considered a significant health care problem, and it is considered the second most cause of death. According to WHO, environmental factors are responsible for more than 70% of cancer cases. Many heavy metals are considered carcinogenic according to the classification of WHO and the International Agency for Research on Cancer (IARC), e.g., cobalt, mercury, lead, arsenic, nickel, cadmium, beryllium, chromium and others [16]. For example, many studies showed that people who drink water containing high levels of arsenic have higher risks of bladder, kidney, lung, colon, liver and skin cancer [17,18]. In addition, cadmium is known to cause kidney, prostate and lung cancer, aluminum can cause lung and bladder cancer [16].

As a first step of water pollution prevention, accurate and rapid monitoring of the heavy metals is vital. Ideally monitoring methods are expected to identify point sources of pollutants and the variation of non-point sources of pollutants in the environment. One of the main sources of heavy metals in harvested rainwater collected from roof surfaces is the roofing material. Storm water runoff, collected from both roof surfaces and ground, collects a variety of pollutants, e.g., excess nutrients, metals, hydrocarbons and pesticides that may leach from traditional roofing materials or may be introduced onto roofs through wet and dry depositions [19,20]. The first flush of runoff water occurring at the beginning of the storm contains a high proportion of the pollutants loads, including heavy metals [21]. Other sources of heavy metals are mineral particles from ground surface, components originating from industrial emissions, vehicles emissions and fuel combustion products emitted to the atmosphere [22,23].

Rainwater harvesting is a common practice in the West Bank of Palestine, especially in the southern part where water is very scarce [24]. In these areas, during winter season, rainwater is collected from the rooftops of the houses and stored in cisterns. There is a high probability that this water might be contaminated with heavy metals coming from, for instance, from dust, roof materials, etc. [25]. Problems related to rainwater quality and its contamination with heavy metals are significant from the point of view of health. While scientific knowledge is important for knowledge-based policy development, combining science and local knowledge from stakeholders is necessary for developing more inclusive approaches and locally targeted solutions [26].

Harvested rainwater, used for domestic purposes including human consumption, cooking, toilets and showers etc. in the southern part of the West Bank, was the main focus of this research. Rural areas in Hebron, specifically different neighborhoods of Yatta area, were selected as the study area, since many of them are not served with water distribution networks, experiencing a shortage of water, and thus resorting to rainwater harvesting techniques [27]. This research brings new cognitive and practical conclusions from the Palestine to science to ensure the safe use of the limited water resources. It concerns both rainwater and surface and groundwater. The research addresses the important issue of evaluating the safety of harvested rainwater. This issue is timely as climate change is impacting freshwater resources and an understanding of the factors that impact contaminant levels is urgently needed. this paper adds to the knowledge pool of water quality, especially rainwater, in water stressed countries of arid to semi-arid climates. The main objectives of this research were to investigate the occurrence of different heavy metals (Pb, Cr, Mn, Co, Ni, Cu, Zn and Cd) in harvested rainwater collected in cisterns; to identify sources of heavy metals pollution of the harvested rainwater; and to assess the potential health risks due to contamination of harvested rainwater used for drinking by heavy metals.

## 2. Methodology

### 2.1. Study Area

**Figure 1:**
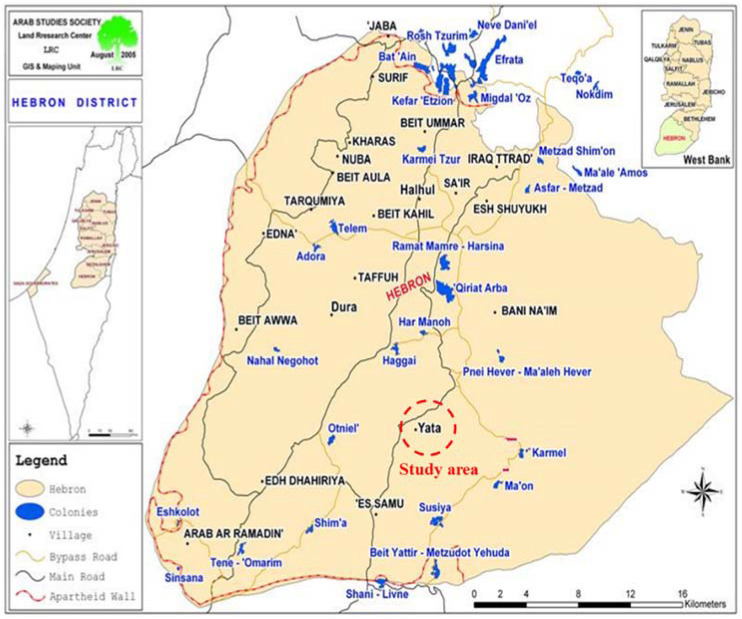
Hebron district including the study area, (PCBS, 2020).

The focus of this research is on Yatta area. Yatta town is approximately 8 km south of the city of Hebron. Hebron is a Palestinian city, located in the southern West Bank, 30 km south of Jerusalem. It lies 930 m above sea level. It is the largest city in the West Bank and the second largest in the Palestinian Territories after Gaza. Hebron is attached to cities of Adh Dhahiriya, Dura, Yatta, and the surrounding villages with no borders. In 2020, its population reached 68,094 capita [28]. Figure 1 shows the Hebron District Map.

Hebron District climate ranges from arid to semiarid with an increase in aridity towards the Negev Desert in the south and the Jordan Valley in the east. The monthly average temperature ranges from 7.5 to 10 °C in winter to 22 °C in summer. The minimum temperature is −3 °C in January and the maximum is 40 °C in August. Most of the rainfalls are during December through February, although there may be rain from mid-October to the end of April. The number of rainfalls per month ranges between 400 mm during the rainfall season and 0 mm during the dry season. Hebron District is facing amplified water scarcity due to the arid and semiarid climatic conditions [29].

### 2.2. Water Sampling 

In this case, 74 water samples were randomly collected from five different neighborhoods in Yatta area, with estimated 75,000 inhabitants having around 4500 cistern. The samples were collected during the rainy season in February 2016 over a period of 5 days. One-time water sample was collected from each of the 74 cistern, and the water samples distribution was as follows: 12 samples from Al-Hila village, 21 from Yatta town, 13 from Khallet Saleh village, 13 samples from Khallet al Maiyya village, and 15 from Al-Hadidya village. First, the water samples were collected in a 1-L high density polyethylene bottles; pre-cleaned with 10% nitric acid followed by repeated rinsing with bi-distilled water, stabilized with ultrapure nitric acid (0.5% HNO_3_), preserved in a cooler of about 4 °C and transported to the lab of Al-Quds University for analysis. The samples were analyzed for heavy metal content (Pb, Cr, Mn, Co, Ni, Cu, Zn and Cd) using Inductively Coupled Plasma Mass Spectrometry (ICP-MS). Preparation of samples was made by diluting 1.0 mL of the water samples to 10.0 mL with 0.3% ultrapure nitric acid. After that, the samples were analyzed by ICP-MS. ICP-MS (Agilent 7500) with an onboard peristaltic pump, a nebulizer (MicroMist nebulizer), an ICP argon plasma torch, two pumps for evacuation, a quadruple mass analyzer, an octapole reaction system (ORS), and an electron multiplier detector was used for the analysis of the heavy metals in this study.

ICP-MS is a type of mass spectrometry that is capable of detecting several trace metals and non-metals at concentrations as low as one part in 10^15^ (part per quadrillion, ppq) on non-interfered low-background isotopes. The detection is achieved by ionizing the sample with inductively coupled plasma first and then using a mass spectrometer to separate and quantify the ions [30]. The results of heavy metal concentrations in the analyzed samples were compared with the Palestinian standards and WHO guidelines for drinking water, as well as the values mentioned in some other developing countries that use harvested rainwater for domestic purposes.

### 2.3. Human Health Risk Assessment

As the harvested rainwater, in the study area, is to be used for drinking and other domestic purposes, it is important to make sure that this water is safe to be used by consumers.

Two approaches were used to test the safety of the harvested rainwater [4]:

1. Chronic daily intakes of metals (CDIs) and,
2. Health risk indexes of metals (HRIs).

The health risk assessment was made for all the samples that exceeded either the WHO or the Palestinian standards for drinking water quality or both.

#### 2.3.1. Chronic Daily Intakes of Metals (CDIs)

Heavy metals enter the human body through several pathways including: food intake, dermal contact and inhalation. In comparison with oral intake, however, all other pathways are considered negligible [31]. The *CDI* (μg/(kg.day)) of a heavy metal through water ingestion was calculated by Equation (1) [31,32].

(1) $$CDI=\frac{C_{m\;}}{W_{b}}\times I_{w}$$

where, $C_{m\;}$(μg/L) is the heavy metal concentration in water, $I_{w}$ (L/day) is the average daily intake of water (assumed to be 2 L/day for adult and 1 L/day for child), and $W_{b}$ (kg) is the average body weight (assumed to be 72 kg for adult and 32.7 kg for child), respectively [31].

#### 2.3.2. Health Risk Indexes of Metals (HRIs)

To estimate the chronic health risks, HRIs were calculated by Equation (2) [31,32].

(2) $$HRI=\frac{CDI}{RfD}$$

where, the oral toxicity reference dose (*RfD*, μg/(kg.day)) values for Cd, Cr, Cu, Mn, Ni, Pb, Zn and Co are 5 × $\;10^{-1}$, 1.5 × $10^{1}$, 3.7 × $10^{1}$, 1.4 × $10^{2}$, 2 × $10^{1}$, 3.6 × $10^{1}$, 3 × $10^{2}$ and 3 × $10^{1}$, respectively [31,32]. The *HRI* value less than 1 is considered to be safe for the consumers [33].

### 2.4. Household Survey

A structured household survey was conducted with the same households where the water samples were collected. A questionnaire of 19 questions in multiple choice format was designed and distributed for this purpose (Appendix A). The questionnaire included questions about the source of water in the cistern, the frequency of cleaning the roof and the cistern, age of the cistern, shape of the cistern, existence of impurities or algae on the surface and sides of cistern and many others. The main aim of the questionnaire was to relate the results of the sampling analysis by the sources of pollution of the samples to heavy metals. In order to connect them together, statistical analysis was made using the Statistical Science Software Program (SPSS) software version 20 [34]. All the sample results exceeding the WHO and Palestinian limits were considered polluted by heavy metals. Cross tabulation method was used in the statistical analysis in order to determine whether there is a statistical significance between the questions, at a significance level of 0.05; which are considered as possible sources of pollution by heavy metals, and the sample results.

## 3. Results and Discussion

### 3.1. Main Characteristics and Usage of Rainwater Harvesting Cisterns in Yatta Area

The main characteristics of rain fed cisterns in Yatta area are discussed in this section. Considering water collection surface, 77.3% of the households depend on rainwater collected from the roof of the house as a major source of domestic water. During winter, they harvest rainwater in cisterns laid in the backyard of the houses. In addition, 1.3% collect water from the garden or the backyard of the house, and 2.7% collect rainwater from the streets. 

The age of cistern varies between the households, 56.4% of the households indicated that their cistern’s age is less than or equal 20 years, while 20.5% had older cisterns with an age of more than 30 years. The age of the cistern can be linked to the quality of the water collected inside cistern. The older the cistern, the higher the probability that it contains impurities and accumulates heavy metals, and therefore the harvested rainwater might become contaminated inside the cistern. The volume of the rainfed cisterns of the households varies between a few to 200 cubic meters, but 34.8% of the households have a cistern of 40 cubic meters or less. 81.3% of the cisterns are made from concrete, while only 14.7% are made from rock. 

The acidic components of rainwater react with the alkaline components of concrete cisterns or cement mortar, dissolving mineral salts (mainly calcium carbonate). Therefore, using harvested rainwater in concrete cisterns might affect its quality. Regarding the cistern top, most of the cisterns (98.6%) have a cover. Closing the cisterns totally guarantee that no or little impurities and heavy metals may enter into them. 61.3% of the households have a cuboid cistern shape; while 38.7% have a peer-shaped cistern. 61.3% of respondents use the roof of the house for hanging the laundry. 

Most of the households (98.7%) use the harvested rainwater for drinking, while 62.7% and 61.3% of them use it for irrigation and cleaning and laundry. Since harvested rainwater is used for many purposes, it is crucial to guarantee that the harvested rainwater is safe for the consumers, i.e., in case of the presence of heavy metals, they should be in minimal concentrations or concentrations below local and international limits, also, the CDI and HRI must be within the international limits to consider the water safe for daily intake. 

With respect to the environmental conditions regarding the rainwater harvesting cisterns, the distance between the cesspit and the cistern in most of households (86%) was 15 m or less. The greater the distance between the cesspit and the cistern, the lower the probability of wastewater leakage from the cesspit into the cistern [35]. 48.5% of the households have the level of cesspit below the cistern level; while 32.4% have the level of cesspit the same level as cistern. It is better that the level of cesspit be lower than the cistern level to prevent wastewater leakage from the cesspit into the cistern. 93.8% of the households emptied the cesspits within the last year. 4.8% of the households emptied cesspits within 3 years and 1.6% within 15 years. Emptying the cesspits periodically guarantees that the wastewater leakage from these cesspits into the cisterns is kept as minimum, therefore making sure that the quality of the harvested rainwater is kept at its maximum level. Most of the households (74.7%) do not have trees close to the cistern, while 25.3% have trees around the house and close to the cistern. Heavy metals, coming from streets through the air, can adsorb to these trees and then might desorb and enter the cisterns polluting the harvested rainwater.

### 3.2. Cisterns Owners’ Awareness

The indicators of cisterns owners’ awareness to the factors that contribute to pollution prevention in rainwater harvesting cisterns are presented in this section. In this case, 96% clean the roof and 81.3% remove the first rain before start collecting rainwater in the cistern. Cleaning the roofs guarantees that no or little amounts of heavy metals can present in the harvested rainwater and that is the case in this study. 75.3% of the households raise animals and birds around the house. Raising animals or birds in the backyard of the house increases the opportunity that these animals pick the heavy metals from streets and therefore these heavy metals may enter the harvested rainwater affecting its quality. 70% of the households clean the cisterns periodically before harvesting the rainwater. Cleaning the cisterns from time to time guarantees that no or very little amounts of heavy metals will be stuck on their walls and this keeps the low concentrations of the heavy metals analyzed in the samples. 77.3% of the households allow solid wastes accumulate in the yard of the house. Solid wastes include many components that may contain heavy metals, and so collecting the waste in the backyard of the house increases the opportunity of contamination of the harvested rainwater with a number of heavy metals such as Pb and Cr coming, for example, from batteries.

### 3.3. Heavy Metals’ Concentrations

The results of the investigated heavy metals concentration in the harvested rainwater are presented in Table 1. Out of the eight tested heavy metals, Cr, Ni and Cu were detected in all samples. Pb, Mn, Co, Zn and Cd were detected in 71, 60, 40, 68 and 46 out of 74 samples, respectively. The results of the current study are compared against international and local studies on the occurrence of heavy metals in harvested rainwater (Table 2). The heavy metal presented in Table 1 and Table 2 are discussed separately hereafter.

Cr. The water samples are considered safe in terms of the occurrence of the heavy metal Cr, except two samples that exceeded the WHO and Palestinian limits (Table 1 and Table 2). Possible sources of Cr are road dust, asbestos brakes or anthropogenic activities occurring around the house [36,37]. In addition, Cr is emitted from solid waste, fossil fuel combustion and steel industry [38,39]. Indeed, several Chromium leather tanning factories exist in Hebron [40]. However, no heavy industrial or nuclear activities are occurring in the study area, and this explains the low value of this metal in the harvested rainwater samples of this research.

Mn. The harvested rainwater samples in the all the studies are considered safe in terms of the occurrence of Mn. In the study area, none of the samples exceeded the WHO and Palestinian limits for this metal. Based on the literature, a possible source of Mn is dust transported through wind to the roofs of the houses [41].

Co. The heavy metal Co occurred in very low amounts in the study areas that were lower than the acceptable limits for drinking water set by the WHO and the Palestinian standards institution (Table 1 and Table 2). Possible sources of Co in harvested rainwater are uncontrolled incineration of solid wastes in illegal dumping sites, vehicles’ exhausts and leakage from engines, pesticides, and sand, soil, silt and others [41].

Ni. The occurrence of the metal Ni in the majority of the samples was far below the WHO and the local Palestinian limits (Table 1 and Table 2). Possible source of Ni is leaching from metals in contact with harvested rainwater such as pipes and fittings which are used to collect the harvested rainwater [36,42]. For this research, the statistical analysis showed that there is a relationship between the source of the water in the cistern and the high level of Ni in two samples. The chemical reactions occurring between the roofing material or pipe with the harvested rainwater may leach out many chemicals including nickel [21,43].

Cu. None of the harvested rainwater samples in this study contained the heavy metal Cu in concentration above the WHO or the Palestinian limits. Possible sources of Cu in harvested rainwater are vehicles’ exhausts, pesticides and industrial activities [39,41,44].

Zn. The metal Zn concentrations, in all the tested samples, were below the Palestinian limits, but only one sample exceeded the WHO limit (Table 1 and Table 2). Based on the questionnaire results and literature, possible sources of Zn are the roof and storage tanks. Rainwater can dissolve the heavy metal Zn and other impurities from materials of the catchment and storage tank. Other source of Zn is the distribution system (pipes) and plumbing, as the pipes are used to collect harvested rainwater from the roofs [36].

Cd. All of the tested harvested rainwater samples were considered safe in terms of the heavy metal Cd. Possible sources of this metal in harvested rainwater are vehicles’ exhausts, leakage from engines, ashes and dust containing Cd transported through wind to roof of houses, pesticides and others [41].

Pb. The water samples are considered safe in terms of the occurrence of the heavy metal Pb, except two samples that exceeded the WHO and Palestinian limits (Table 1 and Table 2). Based on the questionnaire results and literature, possible sources of Pb are the roof and storage tanks. Elevated levels of Pb could be from leaching from metallic roofs and storage tanks or from atmospheric precipitation. In addition, municipal solid waste incinerators are a major source of Pb [45], because of the use of their metal oxides as pigments, stabilizers and catalysts in plastic processing [44].

**Table 2:** Concentration of the heavy metals in harvested rainwater in several countries, as well as the WHO and Palestinian limits.

Element	^1^ Palestine	^2^ Palestine	^3^ Palestine	^4^ Turkey	^5^ Pakistan	^6^ Iran	^7^ WHO Limits	^8^ Palestinian Limits
Cr	4.31	56.1	0.008	4.40	3.61	1.74	50	50
Mn	4.32	112.6	0.003	3.98	14.01	n.a.	500	100
Co	0.24	3.16	n.d.	n.d.	0.52	n.a.	10	n.a.
Ni	19.06	26.7	0.003	3.82	4.25	7.14	70	50
Cu	8.18	143.6	0.003	6.01	65.8	21.4	2000	1000
Zn	201.34	111.8	0.05	6.12	34.2	80.93	3000	5000
Cd	0.10	1.17	n.d.	n.a.	0.53	0.67	3	3
Pb	1.80	45.8	n.d.	n.a.	5.03	69.7	10	10

^1^, This study; ^2^, [42]; ^3^, [46]; ^4^, [47]; ^5^, [31]; ^6^, [48]; ^7^, [49]; ^8^, [50]. All concentrations are in μg/L; n.d.: not detected; n.a: not available.

### 3.4. Factors and Activities Contribute to Harvested Rainwater Contamination with Heavy Metals

To study the effect of the various factors surrounding the well and the activities undertaken by well owners to reduce or prevent water pollution with heavy elements, cross tabulations between these factors and activities, and the four heavy elements polluting water (Table 1): Pb, Cr, Ni and Zn was conducted. Table 3 shows a summary of these cross tabulations. It can be noticed that there are only two statistically significant relationship (*p* < 0.05) between various factors surrounding the well and the activities undertaken by well owners to reduce or prevent water pollution with four heavy elements: the factor “Type of water collection surface” with the metal Ni and the factor “Actions taken before collecting rainwater” with the metals Pb and Cr. Possible source of Ni is leaching from metal pipes and fittings which are used to collect the harvested rainwater [36,42]. Pb could be from metallic roofs and storage tanks. In addition, municipal solid waste incinerators are a major source of Pb [44,45], because of the use of their metal oxides as pigments, stabilizers and catalysts in plastic processing [43,44]. Chromium could had been originated from leather tanning factories [39,40] (see Section 3.3).

### 3.5. Health Risk Assessment

The CDI and HRI values calculated for the meals exceeded the WHO and the Palestinian standards (Table 4) show that the harvested rainwater is safe for consumers (Table 5). This makes sense, as the samples exceeded the limits by small amounts only.

The results of this study show that heavy metals in the harvested rainwater in the study area were not a big issue, as their concentrations were usually below the local and international limits. Most of the studies of in the international literature focus on testing many parameters in drinking water including physiochemical parameters (pH, electrical conductivity, turbidity, alkalinity, hardness, calcium, magnesium and many others) and microbiological parameters (Fecal coliform and Total coliform). For example, a study in the area of Tulkarm in the West Bank, which considered 12 rural areas, focused on these parameters, but the heavy metals were not considered. In general, rural areas do not have heavy industrial nor anthropogenic activities that may produce high amounts of heavy metals such as Pb, Cr and Zn. On the contrary, in the study carried in Tulkarem area, high percentage of the samples were contaminated with Total coliform and Fecal coliform of 86% and 80%, respectively [46]. Therefore, it can be concluded that more attention must be given to the other water quality parameters, especially microbial quality of harvested and drinking waters, especially in rural areas in Palestine. In addition, a one major limitation of this study is that only one sample was collected from each study site which means that the samples collected might not be representative of the true heavy metal levels, and so more samples are recommended to be analyzed. The study did not include analysis of rainwater before it was collected, but this could be considered in a further research.

## 4. Conclusions

This study only presents results for heavy metals but there are several other important contaminants that must be considered for drinking water safety. So, concerning heavy metals contamination, the results of this research showed that most of the water samples analyzed were safe. For the metals, Pb, Cr and Ni; 97.3% of the samples were below the WHO and Palestinian limits, for the metals, Mn, Co, Cu and Cd; none of the samples exceeded the WHO nor the Palestinian limits. For the metal, Zn, 98.7% of the samples were below the WHO limits and none of them exceeded the Palestinian limits.

Statistical analysis showed that there was no relationship between the factors that may lead to contamination with heavy metals and the laboratory results except for the metal Ni and the “type of water collection surface” factor and for the metals Pb and Cr and “Actions taken before collecting rainwater” factor.

Health risk assessment was made for all samples that exceeded the WHO and Palestinian limits. However, and as the metals’ concentrations, which exceeded the limits, exceeded them by small amounts, the samples were considered all safe. This means that the harvested rainwater in the Palestinian rural areas imposes no health risks on consumers, whether they were adults or children, and so can be safely consumed for dinking and other domestic use regarding heavy metals. 

## Figures and Tables

**Table 1:** Concentrations of the heavy metals for all of the samples analyzed in harvested rainwater, Yatta area.

Element	Average (STD) (μg/L) (SD)	Samples Where Each Heavy Metal Was Detected, *n* (%)	Range (μg/L)	WHO Limits (μg/L)	Palestinian Limits (μg/L)	Samples Exceeding WHO Limits, *n* (%)	Samples Exceeding Palestinian Limits, *n* (%)
Pb	1.80 (1.49)	71 (96)	n.d.–24.00	10	10	2 (2.70)	2 (2.70)
Cr	4.31 (3.69)	74 (100)	0.11–101.00	50	50	2 (2.70)	2 (2.70)
Mn	4.32 (2.99)	60 (81)	n.d.–58.00	500	100	0 (0)	0 (0)
Co	0.24 (0.66)	40 (54)	n.d.–3.00	10	-	0 (0)	0 (0)
Ni	19.06 (8.01)	74 (100)	0.60–518.00	70	50	2 (2.70)	2 (2.70)
Cu	8.18 (3.99)	74 (100)	0.54–123.00	2000	1000	0 (0)	0 (0)
Zn	201.34 (21.04)	68 (92)	n.d.–3453.00	3000	5000	1 (1.35)	0 (0)
Cd	0.10 (0.56)	46 (62)	n.d.–2.00	3	3	0 (0)	0 (0)

n.d.: not detected.

**Table 3:** Cross tabulation between the factors that may lead to contamination with heavy metals and the four heavy elements polluting water: Pb, Cr, Ni and Zn.

Factor	Significance *p*-Value			
	Zn	Ni	Cr	Pb
Type of water collection surface	1.000	0.000	0.996	0.996
Take actions before collecting rainwater (Cleaning the roof of the house, Getting rid of first rain water, etc.).	0.972	0.925	0.000	0.000
For what purposes is the water in the cistern used	0.977	0.996	0.983	0.983
When the cistern was cleaned last time?	1.000	0.997	0.997	0.997
Walls material of cistern	0.905	0.817	0.817	0.817
Situation of the cistern top	0.906	0.867	0.867	0.867
Shape of the cistern	0.424	0.739	0.255	0.255
Distance between cistern and cesspit	1.000	1.000	1.000	1.000
Level of cesspit with respect to cistern	0.192	0.230	0.230	0.230
When the cesspit was discharged last time	0.999	0.996	0.996	0.996
Is there any animals or birds raised in the house	0.443	0.714	0.714	0.714
Is there any trees close to the cistern	0.558	0.404	0.404	0.404
Do you use the roof of the house for hanging the laundry	0.424	0.255	0.255	0.255
Do you allow solid waste to accumulate in cistern vicinity	0.586	0.438	0.349	0.349

**Table 4:** Summary of the health risk assessment calculations (for adults).

Heavy Metal	Number of Sampled that Exceeded the Standard Drinking Water	CDI Value (μg/kg.day)	HRI Value	Result
Pb	1	0.668	0.019	Safe
	2	0.382	0.011	Safe
Cr	3	2.82	0.188	Safe
	4	1.70	0.113	Safe
Ni	5	5.97	0.30	Safe
	6	14.39	0.72	Safe
Zn	7	95.90	0.32	Safe

**Table 5:** Summary of the health risk assessment calculations (for children).

Heavy Metal	Sample No.	CDI Value (μg/kg.day)	HRI Value	Result
Pb	1	0.735	0.020	Safe
	2	0.421	0.012	Safe
Cr	3	3.10	0.207	Safe
	4	1.87	0.125	Safe
Ni	5	6.57	0.33	Safe
	6	15.84	0.79	Safe
Zn	7	105.60	0.35	Safe

## Data Availability

The datasets generated during and/or analyzed during the current study are available and can be obtained by contacting the corresponding author.

## Appendix A. Questionnaire

Q 1	- What is the source of water in cistern? 1. Roof of the house 2. Garden or the backyard of the house 3. Street 4. Otherwise (indicate)
Q 2	- Do you take any actions before collecting the rainwater? 1. Yes 2. No
Q 3	- If the answer of Q 2 is Yes, what are these actions? 1. Cleaning the roof of the house 2. Getting rid of first rain water 3. Both 1 and 2 4. Otherwise (indicate)
Q 4	- When the cistern was cleaned last time? Before:
Q 5	- For what purposes is the water in the cistern used? (The answer may be more than one choice) 1. Drinking 2. Animals 3. Irrigation 4. Cooking 5. Cleaning the house 6. Otherwise (indicate)
Q 6	- Indicate approximately the age of cistern in years
Q 7	- What is the volume of the cistern
Q 8	- The walls of the cistern are from: 1. Concrete 2. Rock 3. Otherwise (indicate)
Q 9	- Is the cover of the cistern: 1. Open 2. Closed 3. Perforated
Q 10	- The shape of cistern: 1. Cuboid 2. Peer-shaped 3. Otherwise (indicate)
Q 11	- The distance between cistern and cesspit (in meters)
Q 12	- The level of cesspit: 1. Above cistern level 2. Below cistern level 3. Same level as cistern
Q 13	- When the cistern was discharged last time? Before
Q 14	- Do you raise animals or birds around the house? 1. Yes, always 2. Sometimes 3. No
Q 15	- Is there any trees close to the house? 1. Yes 2. No
Q 16	- Do you notice any impurities on the surface of the water in the cistern? 1. Yes 2. No
Q 17	- Do you notice algae on the sides of the cistern? 1. Yes 2. No
Q 18	- Do you use the roof of the house in winter for hanging the laundry? 1. Yes 2. No
Q 19	- Do you collect solid waste in the backyard of the house? 1. Yes 2. No
